# Nutrition and aroma challenges of green tea product as affected by emerging superfine grinding and traditional extraction

**DOI:** 10.1002/fsn3.1768

**Published:** 2020-07-09

**Authors:** Pai Peng, Linlin Wang, Guowei Shu, Jianke Li, Li Chen

**Affiliations:** ^1^ College of Food Engineering and Nutritional Science Shaanxi Normal University Xi’an P. R. China; ^2^ School of Food and Biological Engineering Shaanxi University of Science and Technology Xi’an P.R. China

**Keywords:** antioxidant activity, extraction, green tea, nutrition and flavor composition, superfine grinding

## Abstract

Green tea, superfine green tea powder (SGTP), and tea extract were prepared to determine their chemical components and antioxidant activity. The nutrition and aroma challenges of green tea during traditional extract technique and superfine grinding were profiled in this study. The traditional extract technique took advantage over superfine grinding in L‐theanine and glutamic acid (Glu) preservation, but not in other 16 essential amino acids. SGTP preserved a maximum of elements from green tea, whereas tea extract greatly changed the elements ratio. Tea extract contained higher VB2 and VC contents and doubled the tea polyphenol (TP) content in comparison with green tea and SGTP. Additionally, tea extract contained more favorable aroma compounds and maintained stronger antioxidant activity in comparison with green tea and SGTP. This study profiled an important basis for the comprehensive utilization of green tea resources by consumers and manufacturers.

## Graphical abstract

Demand and competition for utilization of tea resource have transferred the form of the art of drinking tea into the form of eating tea. Over last decades, products that contain tea extract and superfine grinding tea powder are preferred by consumers and become popular in food and cosmetics industry. However, characterization of nutrition and aroma compositions toward green tea, tea extract, and superfine grinding tea powder is scarce. In this study, the nutrients (polyphenols, amino acids, vitamins, and elements) and aroma composition among different forms of green tea products are investigated, paving the new way to deep processing and high‐value utilization of green tea resources.

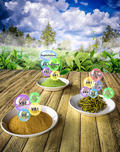

## 1 INTRODUCTION

Tea is one of the most popular consumed beverages in the world. Recently, a new generation of middle‐class consumers is becoming more adventurous in their tea‐drinking habits and driving value growth (Tea in, 2018: Annual market overview, 2018). A growing body of evidence continues to emerge demonstrating a variety of potential health benefits from consumption of green tea and its constituents (Cao et al., 2019; Meng et al., 2019; Nagao, Hase, & Tokimitsu, 2007; Xu et al., 2019). Tea polyphenols, the main composition of green tea, contribute to the sweetness, bitterness, and astringency (Yao et al., 2006). Amino acids, accounting for 3%–4% of green tea, are in charge of the briskness taste (Hara, Luo, Wickremashinghe, & Yamanishi, 1995). Other active constitutes, such as caffeine and water‐soluble carbohydrates, have been investigated to contribute to the bitterness and sweetness aftertaste (Hu, Chen, & Ni, 2012). Additionally, green tea also maintains various vitamins (e.g., C, E, and B) and trace elements (e.g., Ca, Mg, and Zn) to the diet (Hara et al., 1995). These components are essential quality parameters to evaluate green tea quality (Khokhar & Magnusdottir, 2002).

There is growing consumer interest in the green tea material supplemented products in recent years. The demand and competition for utilization of tea resource have transferred the form of the art of drinking tea into the form of eating tea, such as green tea cake, green tea noodle, and green tea bread and so on. Japanese sweets, such as mochi rice cakes and green tea ice cream, are flavored with the green tea.

Nowadays, the industrial utilization of green tea is mainly in the form of tea extract and tea powder. Green tea with traditional extract technology is standardized in the production phase to guarantee that a certain percentage of the active ingredients remains but resulting in more than 40% soluble solids wasted in spent leaf (Xiao, Zhang, Fan, & Han, 2017). Superfine green tea powder (SGTP) is created by increasing tea particle surface area and the breakdown of the cell walls at a low temperature (Zhao, Yang, Gai, & Yang, 2009; Zhu, Huang, Peng, Qian, & Zhou, 2010). High‐grade forms of green tea powder often produce a creamy texture and savory taste, such as Japanese matcha, which contains several nutrients, including vitamins A and C. Xiao et al. (2017) significantly raised infusion yield of total polyphenols, caffeine, and water‐soluble carbohydrate of black tea by superfine grinding technique (Xiao et al., 2017).

Aroma is an important factor for the evaluation of the green tea quality and has been focused in tea science for a long time. So far, more than 600 volatile compounds have been identified in tea (Yang, Baldermann, & Watanabe, 2013; Zhu et al., 2016). A small change in the potent aromatic components may lead to a great difference in tea flavor. Over the past decades, products that contain tea extract and SGTP are preferred by consumers and become popular in food and cosmetics industry. However, the characterization of nutrition and aroma compositions toward green tea, tea extract, and SGTP is scarce.

In this study, we compare the chemical composition of green tea products made by traditional extraction and emergent superfine grinding technique. The nutrients (polyphenols, amino acids, vitamins, and elements) and aroma composition among different forms of green tea products are investigated, paving the new way for deep processing and high‐value utilization of green tea resources.

## 2 MATERIALS AND METHODS

### 2.1 Samples and chemical reagents

Green tea (provided by Shaanxi wo‐long tea manufacture Co., Ltd) was ground by planetary ball mill MITR‐XH‐XQM (Mitrcn Co. Ltd, Changsha, China) at 4°C for 2 hr to obtain SGTP. The particle size of SGTP was measured by a BT‐9300ST laser particle size distribution instrument (Baite Co. Ltd, Dandong, China) with water solvent at room temperature. The median diameter (D_50_) of SGTP was 9.215 μm which indicated the cell wall breakage ratio Φ was 100%. The particle size of SGTP was observed by *SEM* at 5,000 × magnification (Figure S1). Vascular bundles, leaf veins, and threaded conduit structures barely were observed.

Green tea extract was prepared according to our previous study (He et al., 2015). Green tea was dissolved into 50% ethanol (v/v) at a ratio to 1:25 (w/v) and heated at 80°C for 20 min. Then, tea extract was obtained. All the reagents were of analytical, HPLC, or GC‐MS grade and purchased from Sigma‐Aldrich (St. Louis, MO, USA).

### 2.2 Analysis of amino acids

#### 2.2.1 Determination of L‐theanine by HPLC

3.0 g samples (green tea, tea extract and SGTP) were prepared and dissolved into 10 ml ultrapure water for ultrasonic extraction 15 min at 80 Hz, respectively. Then, the ultrasonic extraction was repeated twice in a 5 min interval. The supernatants were combined and stored at 4°C in darkness. L‐theanine was analyzed by HPLC under the following conditions. Agilent ZORBAX SB‐C18 (5 μm, 4.6 × 250 mm) combines a mixture solution (ratio of ammonium acetate, methanol, and acetonitrile is 1:2:2, v/v/v) and ammonium acetate (20 mmol/L) at a ratio to 3:7 (v/v) as the mobile phase at a flow rate of 1 ml/min. Column temperature is fixed at 25°C, UV detection wavelength is 338 nm, and sample size is 20 μL. Before injection, 1 ml OPA derivative reagent was added into the samples, respectively. Then, the mixtures were sealed at room temperature for 2 min and filtrated through 0.45 μm inorganic membrane.

#### 2.2.2 Determination of essential amino acids by amino acid analyzer

The amino acid composition was identified and quantified by the equipment amino acid analyzer L‐8900 (Hitachi, Tokyo, Japan). 1.0 g samples (green tea, tea extract and SGTP) were hydrolyzed in 10 ml of 6.0 M HCl, microwaved for 2 min and flushed with nitrogen at 110°C for 23 hr, respectively. After nature cooling, the samples were filtered through a 0.45‐μm syringe filters.

### 2.3 Analysis of water‐soluble vitamins: vitamin C (VC), vitamin B1 (VB_1_), vitamin B2 (VB_2_), and folic acid

#### 2.3.1 HPLC determination of VC

The chromatographic conditions of VC were shown as follows. Besides Agilent Eclipse Plus‐C18 column (5 μm, 4.6 × 250 mm), a mixture of 0.01 mol/L K_2_HPO_4_ (PH 2.5) and methanol at a ratio of 98:2 was used as mobile phase at a flow rate of 0.7 ml/min. Column temperature is 25°C, DAD range is 245 nm, and sample size is 20 μL, respectively.

#### 2.3.2 HPLC determination of VB_1_ and VB_2_

The chromatographic condition of VB_1_ was as follows: Dikma Diamonsil C18 column (5 μm, 4.6 × 150 mm); a mixture of 0.05 mol/L NaAc (PH 4.5) and methanol at a ratio of 65:35 was used as mobile phase at a flow rate of 0.8 ml/min; column temperature: 35°C; FLD: Ex: 375 nm, Em: 435 nm; and sample size: 20 μl.

The chromatographic condition of VB_2_ was similar to the above‐mentioned except the below information. The flow rate of 1 ml/min, column temperature of 30°C, and FLD with Ex of 462 nm and Em of 522 nm are fixed.

#### 2.3.3 HPLC determination of folic acid

The chromatographic condition of folic acid was shown below: Agilent Eclipse Plus‐C18 column (5 μm, 4.6 × 250 mm); a mixture of 0.1 mol/L KH_2_PO_4_ and acetonitrile was used as mobile phase at a flow rate of 1 ml/min; column temperature: 35°C; DAD range: 260 nm; and sample size: 10 μl.

### 2.4 Analysis of trace elements

The elements of tea samples were detected by X‐ray fluorescence spectrometer EDX‐7000 (Tokyo, Japan) under air atmosphere and 1 cm collimator. The content of element is expressed in atomic percentage.

### 2.5 Analysis of aroma components by SPME‐GC‐MS

1.0 g samples (green tea, tea extract, and SGTP) were dissolved into 5 ml boiling ultrapure water in SPME (Supelco, Bellefonte, USA) tube and sealed, respectively. DVB/CAR/PDMS fiber was suited to analyze the aroma emitted from tea. The samples were balanced in water bath at 70°C for 10 min, then were absorbed by solid‐phase microextraction for 50 min, and finally injected by GC‐MS after 5 min of desorption. Aroma compounds were analyzed on Agilent 7890A‐7000B GS‐MS. The gas chromatograph was equipped with a DB‐225MS capillary column (30.0 m × 0.25 mm × 0.25 μm). The injection was operated with a split ratio of 10:1. The running program was 40°C for 2 min, then ramp the temperature with the rate of 5°C min^‐1^ until 230°C, and held for 8 min. The source temperature of the electron impact mode was 200°C. The ionize voltage was 70 eV. The full scan mode was in range from 29 to 500 amu, and the solvent delay time was 0 min.

### 2.6 Determination of tea polyphenol (TP) and analysis of antioxidant capability

All tea samples were prepared in the same way, dissolved in 50% ethanol at a ratio to 1:25, and heated at 80°C for 20 min. TP was determined according to the National Standard of the People's Republic of China (GB/T 8313–2008) (Chinese National Standard GB/T^8313^, ^2008008^).

Antioxidant capacity of green tea products was evaluated by the methods of 2,2‐diphenyl‐1‐picrylhydrazyl (DPPH) radical scavenging activity, ferric reducing antioxidant power (FRAP), and 2,2′‐azino‐bis (3‐ethylbenzo‐ thiazoline‐6‐sulfonic acid diammonium salt) (ABTS·+) free radical scavenging activity (Chen, Feng, & Zhang, 2009).

### 2.7 Statistical analysis

All the experiments were repeated three times. Significant differences were analyzed by using Duncan's multiple‐range tests with the aid of SAS9.1.3 software (North Carolina, Cary, USA) (*p* < .05).

## 3 RESULTS AND DISCUSSION

### 3.1 The content of L‐theanine and essential amino acids in different forms of tea products

**Figure 1:**
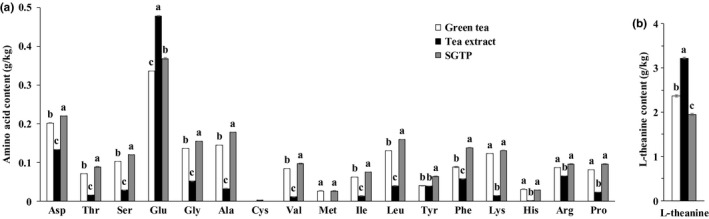
The content of (a) essential amino acids and (b) L‐theanine in green tea, tea extract, and SGTP. L‐theanine was analyzed by HPLC with Agilent ZORBAX SB‐C18 (5 μm, 4.6 × 250 mm). The essential amino acid composition was identified and quantified by an amino acid analyzer L‐8900 (Hitachi, Tokyo, Japan)

Amino acids contribute to main flavor and sweetness in green tea. Of these amino acids, more than 50% are L‐theanine, which is a special kind of amino acids in green tea and is known to act in the brain following oral ingestion (Adhikary & Mandal, 2017; Türközü & Şanlier, 2015). Hu et al reported that there were no significant differences in amino acids of green tea powder with the decrease in particle size during superfine grinding (Hu et al., 2012). Therefore, we focused on the difference of amino acids in tea extract and SGTP. Figure 1 depicts the content of L‐theanine in the form of green tea, tea extract, and SGTP. Tea extract exhibited significant high content of L‐theanine and Glu. This result indicates that the traditional extract technique takes advantage over superfine grinding in L‐theanine and Glu preservation. However, superfine grinding is significantly superior to extract technique to preserve 16 essential amino acids except Glu and Cys. L‐theanine has structural similarity to Glu, with its distinct attribute being a refined, rich flavor, and sweetness. It is believed that most of the sweet taste in green tea is due to L‐theanine. Other amino acids, such as Arg and Ala, also contribute to the bitterness of green tea (Ohtsuki, Kawabata, Kokura, & Taguchi, 1987; Zhu et al., 2016). Therefore, we hypothesized that tea extract remained a better sweet flavor and lower bitterness than of green tea and SGTP.

### 3.2 Elements in different forms of green tea products

**Figure 2:**
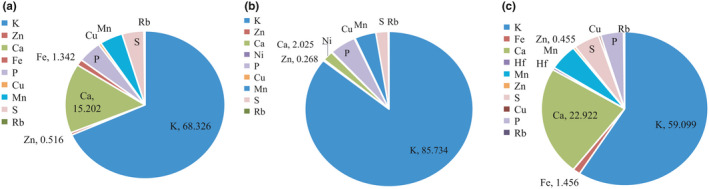
The content of elements in (a) green tea, (b) tea extract, and (c) SGTP. The content of element is expressed in atomic percentage

Tea plays a major role in terms of intake of macro‐ and micro‐nutrients, but also nonessential toxic trace elements may accumulate in tea leaves. Karat and Bhagat (2010) summarized on the concentrations of Al, As, Cd, Cr, Cu, Mn, and Ni in tea leaves and tea infusion (Karak & Bhagat, 2010). A variety of trace elements in tea play an important role in human health, which have contacted with not only the composition itself but extraction rate of trace element in tea. In this study, we investigated the elements composition of three different forms of green tea products with the aid of X‐ray fluorescence spectrometer (Figure 2). The content of elements of green tea products is expressed in atomic percentage.

**Figure 3:**
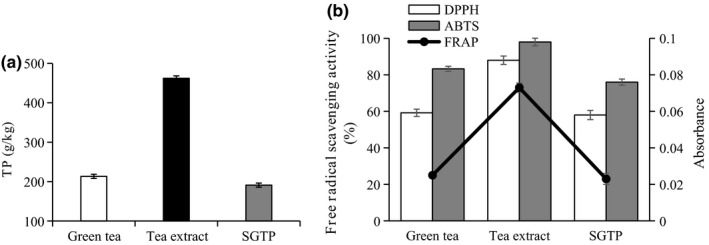
(a) The content of TP and (b) the antioxidant activities of green tea, tea extract, and SGTP. The TP was determined by colorimetric method using Folin–Ciocalteu reagent. The antioxidant activities were valued by DPPH, ABTS, and FRAP

Although Mn is heavy metal in green tea, it is one of the essential trace elements in human body which constitutes several enzymes with important physiological functions. As shown in Figure 3, the content of Mn was 4.668% in green tea, which increased to 5.468% in SGTP but decreased to 4.093% in tea extract. Macro‐elements K, Ca, and P were compared toward the different forms of green tea products. Tea extract contained the highest K content of 85.734%, but the lowest Ca content of 2.025%, when compared with other two products. Superfine grinding significantly increased the Ca content from 15.202% to 22.922% and decreased the K content from 68.326% to 59.099% in comparison with green tea. There was no statistical difference of P content toward three green tea materials. The trace elements of Fe, Zn, and Cu were detected and are shown in Figure 2. There was no statistical significant difference of three trace elements content between the green tea and SGTP. However, the content of Zn and Cu in the form of tea extracts was reduced by half in comparison with the forms of green tea and SGTP. Worse yet, Fe was not detected in tea extract. Superfine grind preserved a maximum of elements from green tea, whereas extract technique greatly changes the elements ratio.

### 3.3 Vitamins in different forms of green tea products

Vitamins in green tea are classified into two families, lipid‐soluble vitamins and water‐soluble vitamins. The latter family contains vitamin C and B, which are absorbed directly in human body through the way of drinking tea (Guo & Yu, 2012). VC, also known as ascorbic acid, can enhance the bioavailability of green tea, and hence its anticancer effects (Mathé, 1999). VB1 (thiamine) is involved in nervous conduction and energy metabolism. It works on the messengers in the brain and supports the transmission of the stimulus. VB2 (riboflavin) supplies the body with energy and spurs the transport of oxygen and gets it to wherever your body needs it the most (Stagg & Millin, 1975). VB9 (folic acid) is important for women during pregnancy, because it helps the body to produce DNA and to form blood. Clinical trials demonstrated that VB9 deficiency may cause malformations of the central nervous system of a fetus (Stagg & Millin, 1975).

Our study compared the content of water‐soluble vitamins in different forms of green tea products with the aid of HPLC (Table 1). The content of VC was the maximum of 133 mg/100 g in green tea, but declined by half in SGTP. The content of vitamin C in green tea extract was 111 mg/100 g, which was lower than that in green tea but significantly higher than that in SGTP. In terms of VB2, green tea extract had the highest VB2 content 0.47 mg/100 g. Green tea and SGTP had significant lower VB2 contents which were 0.115 mg/100 g and 0.105 mg/100 g, respectively. VB1 content was extremely low in all green tea products. VB9 was only detected in green tea, but was not detected in other two forms of green tea products. These results suggested that extract technique significantly increased VB2 content in green tea and took advantage over superfine grind on VC preservation.

**Table 1:** The content of water‐soluble vitamins in green tea materials

	Green tea	Tea extract	SGTP
Vitamin B1 (mg/100g)	＜0.1	＜0.1	＜0.1
Vitamin B2 (mg/100g)	0.115 ± 0.005	0.47 ± 0.028	0.105 ± 0.004
Vitamin C (mg/100g)	133 ± 2.73	111 ± 2.15	76.4 ± 1.03
Folic acid (mg/kg)	＜0.05	ND	ND

Note

ND indicates not detected; the content of water‐soluble vitamins in different forms of green tea products was analyzed by HPLC.

### 3.4 TP content and antioxidant activities of green tea products

The most favorable effect of green tea is accredited to the green tea polyphenol, which is believed to be beneficial to human health (Ong & Annuar, 2017). Depending on how the tea is processed and brewed, the polyphenol level can vary (Das, Kim, Hong, & Eun, 2019). In this study, we determined the content of TP in green tea, tea extract, and SGTP and compared their antioxidant activities (Figure 3). The TP content of SGTP seemed to be lower that of green tea, but not significantly. Hu et al. also reported that the contents of TP in green tea powder decreased with particle size decreasing during superfine grinding. We hypothesized that superfine grinding increased superficial area of green tea powder, and as a result, the contact area with oxygen could be increased largely, which leads to TP oxidation and causes more loss. However, tea extract doubled the TP content which was much higher than that in green tea and SGTP (Figure 3a).

Green tea polyphenols are well‐known for their antioxidant properties. Previous studies have demonstrated that TP is exceptional electron donors and effective scavenger of physiologically relevant reactive oxygen species in vitro (Andrea & Michael, 1997). Figure 3b depicts the antioxidant activities of green tea products in three methods. The DPPH values are varied from 59.2% to 88%, the ABTS values are changed from 83.3% to 98%, and the FRAP values are altered from 0.025 to 0.073, respectively. It was obviously demonstrated that tea extract showed the strongest antioxidant activities than that of two products. There was no significant difference of antioxidant activities between green tea and SGTP. Hu et al. reported that tea polysaccharide increased with the particle size increasing during superfine grinding, which could improve green tea powder antioxidant activity (Hu et al., 2012). The decreased TP content weakens the antioxidant activity theoretically, whereas the increased tea polysaccharide could remedy it. Our results indicated that traditional extract technique took advantage over superfine grinding technique in maintaining antioxidant activity.

### 3.5 Aroma components in different forms of green tea products

**Figure 4:**
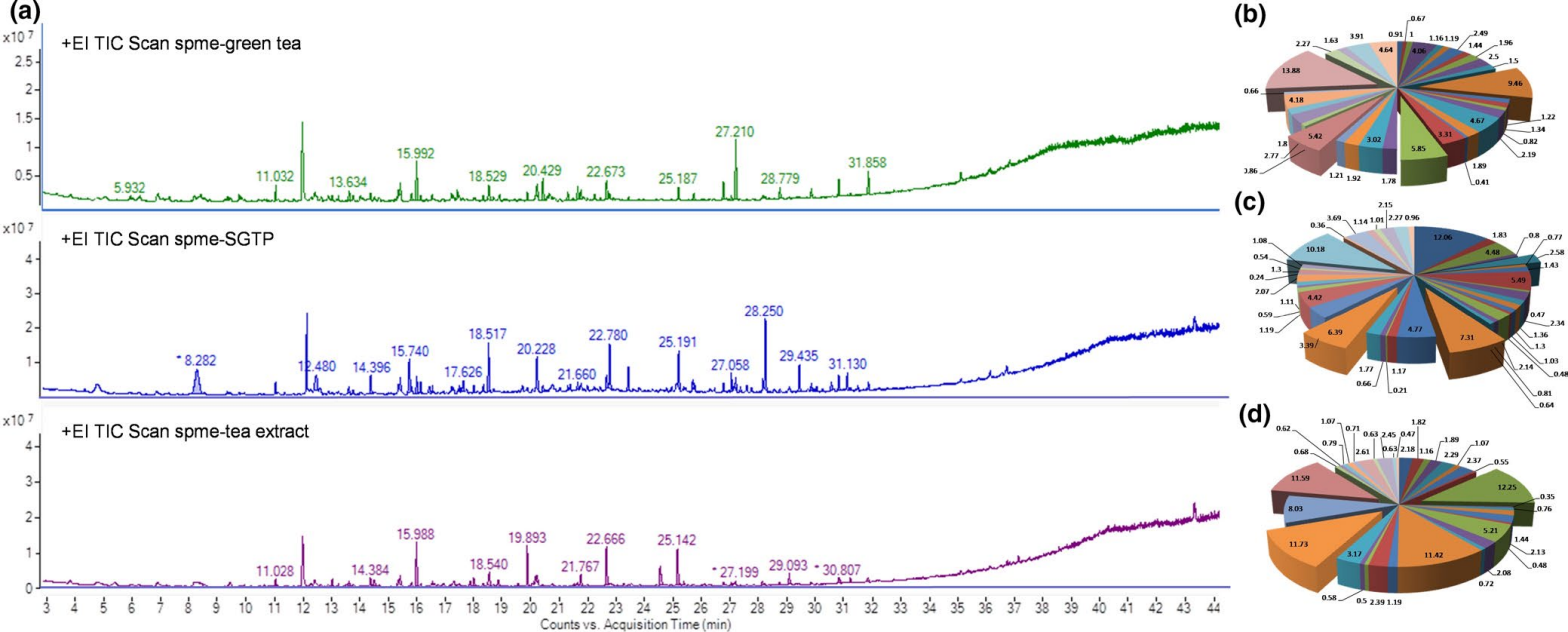
Analysis of aroma compounds in green tea, SGTP, and tea extract. (a) Aroma fingerprint chromatogram by SPME‐GC‐MS; area sum% of aroma compounds composition in (b) green tea, (c) SGTP, and (d) tea extract

Green tea contains high levels of indole pyridine, linalool, geraniol, benzyl alcohol, 2‐phenyl‐ethanol, 2‐ethylhexanoic acid, and maltol, which are important volatiles contributing to the typical aroma of green tea (Alluhayb & Logue, 2017). Some active‐aroma volatiles significantly contribute to the flavor of tea because of their very low odor perception thresholds for human beings (Kumazawa & Masuda, 1999; Lee, Chambers, Chambers, Adhikari, & Yoon, 2013). These key aroma compounds are easily affected by processing technique. We compared the aroma composition of green tea products which were processed by traditional extract and emerging superfine grinding technique. Figure 4a depicts the aroma fingerprint chromatogram of green tea, tea extract, and SGTP, which gives the clues of aroma component changes during different processing. According to the pie chart, we could see the proportion of area sum% of each aroma compound in three green tea products (Figure 4b–d). Aroma compounds which showed top large area sum % were collected and analyzed in Table 2. Green tea contained high level of β‐Ionone, 1,6‐Octadien‐3‐ol, 3,7‐dimethyl‐, β‐cyclocitral and benzaldehyde, 2,5‐dimethyl‐, which were all characteristic aroma flavor in green tea. The top one β‐Ionone, whose area sum % was 13.88%, showed fragrance of violet and raspberry. Followed 1,6‐Octadien‐3‐ol, 3,7‐dimethyl, whose area sum% was 9.46%, sent forth a delicate fragrance of flower. β‐cyclocitral and benzaldehyde, 2,5‐dimethyl‐ give off the fragrance of sweet fruit and almond‐like smell. Tea extract mainly contained 1,6‐Octadien‐3‐ol and 3,7‐dimethyl‐, benzaldehyde, 2,5‐dimethyl‐, whose area sum% were 12.25% and 11.73%, which represented flower and almond‐like smell, respectively. SGTP maintained less aroma compounds, naphthalene, and 1‐Hexanol, 2‐ethyl‐ which smelled of tar, camphoric and greasy odor. According to above data, although green tea products from two different processes well‐maintained aroma flavor, extract technique took advantage over superfine grinding on aroma flavor preservation.

**Table 2:** Main aroma compounds in green tea, SGTP, and tea extract

	Area Sum %	Retention time/min	Characteristic peak (GC‐MS)	CAS No.	Odor quality
Green tea	13.88	27.21	β‐Ionone	79–77–6	Violet, flower, raspberry
	9.46	15.992	1,6‐Octadien−3‐ol, 3,7‐dimethyl‐	78–70–6	flower
	5.85	20.429	β‐cyclocitral	432–25–7	sweet, fruit
	5.42	22.673	Benzaldehyde, 2,5‐dimethyl‐	5779–94–2	Almond‐like smell
SGTP	12.06	8.282	Cyclopentasiloxane, decamethyl‐	541–02–6	
	10.18	28.25	Acenaphthene	83–32–9	
	7.31	18.517	Cyclohexanol, 1‐methyl−4‐(1‐methylethyl)‐	21129–27–1	
	6.39	22.78	Naphthalene, 2‐methyl‐	91–57–6	
	4.77	20.23	Naphthalene	91–20–3	Tar, camphoric and greasy odor
	2.58	14.396	1‐Hexanol, 2‐ethyl‐	104–76–7	flower
Tea extract	12.25	15.988	1,6‐Octadien−3‐ol, 3,7‐dimethyl‐	78–70–6	flower
	11.73	22.666	Benzaldehyde, 2,5‐dimethyl‐	5779–94–2	Almond‐like smell
	11.59	25.142	Cyclododecane	294–62–2	
	11.42	19.893	Cyclohexane, [(1‐methylpropyl)thio]‐	7133–22–4	
	2.61	29.093	Phenol, 2,4‐bis(1,1‐dimethylethyl)‐	96–76–4	pungent

## 4 CONCLUSION

Comprehensive exploration of green tea by traditional extract and emerging superfine grinding technique adds the value of tea materials and positively encourages the deep processing agricultural products. Our study profiled the nutrition and aroma challenges of green tea products processed by traditional extract and emerging superfine grinding. Traditional extract technique took advantage over superfine grinding in L‐theanine and Glu preservation, but not in other 16 essential amino acids. SGTP preserved a maximum of elements from green tea, whereas tea extract greatly changed the elements ratio. Tea extract contained higher VB2 and VC content and doubled the TP content in comparison with green tea and SGTP. Tea extract contained more favorable aroma compounds and maintained stronger antioxidant activity in comparison with green tea and SGTP.

## CONFLICT OF INTEREST

The authors declare no competing financial interest.

## Supporting information

Fig S1
