# Supplementary material for: Nutrition and aroma challenges of green tea product as affected by emerging superfine grinding and traditional extraction
Source: Food Sci Nutr. 2020 Jul 9;8(8):4565–72. doi: 10.1002/fsn3.1768 (PMC7455925; doi:10.1002/fsn3.1768)

**Supporting Information**

Nutrition and aroma challenges of green tea product as affected by emerging superfine grinding and traditional extraction

Pai Peng ^1^, Linlin Wang ^1^, Guowei Shu ^2,*,^ Jianke Li ^1,*^, Li Chen ^1,*^

^1^ College of Food Engineering and Nutritional Science, Shaanxi Normal University, Xi’an 710119, P. R. China.

^2^ School of Food and Biological Engineering, Shaanxi University of Science and Technology, Xi’an 710021, P.R. China

^*^Corresponding Authors

E-mail: chenlisp@snnu.edu.cn (Li Chen)

shuguowei@gmail.com (Guowei Shu)

Jiankel@snnu.edu.cn (Jianke Li)

Tel: +86-29-85310517

**Figure S1.** SEM images for particle size of SGTP in 5000 × magnification.


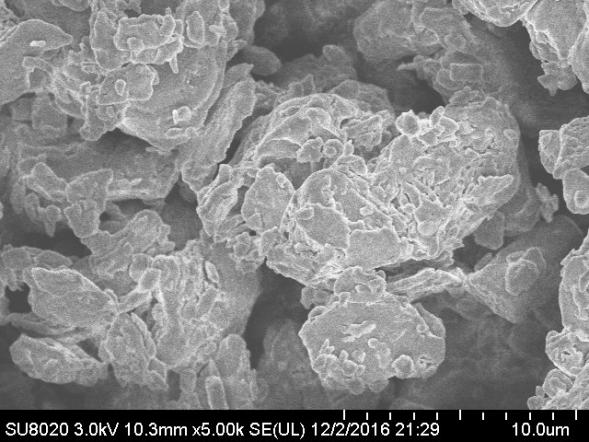

Supplement: Supplementary file 1 — Fig S1 [file FSN3-8-4565-s001.docx]
